# Preserving Biodiversity of Sheep and Goat Farming in the Apulia Region

**DOI:** 10.3390/ani15111610

**Published:** 2025-05-30

**Authors:** Antonella Santillo, Antonella della Malva, Marzia Albenzio

**Affiliations:** Department of Agriculture, Food, Natural Resources and Engineering (DAFNE), University of Foggia, 71121 Foggia, Italy; antonella.santillo@unifg.it (A.S.); marzia.albenzio@unifg.it (M.A.)

**Keywords:** small ruminants, sustainability, autochthonous breeds, biodiversity conservation, Mediterranean countries, traditional dairy products

## Abstract

The interconnection between biodiversity loss, food system and climate change is a complex issue with profound implications for global sustainability. Small ruminant farming is a crucial part of addressing these challenges as it contributes to environmental, social, and economic resilience. In Italy, sheep and goat farming is most common in marginal areas with a prevalence of pastoral systems and low mechanization levels. In the Apulia region of Southern Italy, autochthonous small ruminant breeds are at high risk of extinction, due to changing agricultural practices and market pressures. Autochthonous breeds represent valuable genetic resources, adapted to the local environment and capable of producing high-quality products. Apulia boasts an ancient dairy tradition, producing a variety of cheeses from small ruminants, such as Canestrato Pugliese, a Protected Designation of Origin, and four cheeses (Cacioricotta, Pecorino Foggiano, Scamorza di Pecora, and Caprino) recognized as Traditional Agri-Food Products by the Italian Ministry of Agriculture, Food Sovereignty and Forests. These products represent an essential element for biodiversity conservation, encompassing ecosystems, autochthonous breeds, microbial diversity, traditional farming practices, and production systems. This review surveys the main small ruminant native breeds of Apulia region, highlighting their historical significance, distinctive traits, and traditional productions, to help shape strategies for animal biodiversity conservation.

## Simple Summary

Small ruminants like sheep and goats have adapted over thousands of years to different environments but are now at risk of extinction due to market pressure, loss of traditional practices, and climate change issues. The Apulia region of Southern Italy, with a strong agro-pastoral tradition, is characterized by several sheep and goat autochthonous breeds well adapted to the local environment. This review reports the principal ovine and caprine autochthonous breeds, their historical role, unique traits, and traditional products. A focus on their dairy products, recognized with Protected Designation of Origin and Traditional Agri-Food status, is also addressed. The review stresses the importance of preserving these breeds through genetic characterization, sustainable farming, and promoting traditional products. Protecting local breeds helps safeguard animal biodiversity and landscape conservation, according to the EU Biodiversity Strategy for 2030.

## 1. Introduction

Small ruminant farming represents the most significant agricultural activity in many regions worldwide, contributing not only to economic development but also playing crucial roles in ecological, environmental, and social sustainability [1]. Small ruminants were domesticated over 10,000 years ago and have been farmed in different environments since then for various purposes, including the production of meat, milk, and wool [2,3,4].

Sheep and goats evolved over time in different environments thanks to their ability to cope with extreme conditions, ranging from high mountains to deserts, resulting in the development of different breeds adapted to local environments [5]. However, starting in the second half of the twentieth century, farmers have increasingly replaced traditional, locally adapted livestock breeds with a smaller selection of improved and well-defined breeds that have been intensely selected for specific traits. Advancements in livestock management, including artificial insemination, embryo transfer, crossbreeding, and improved feed technology, have accelerated the diffusion of industrial breeding. As a consequence, the livestock sector in developing countries faces significant challenges, including the progressive decline of rural farming systems and the loss of autochthonous breeds heritage [6,7,8]. In addition to these pressures, climate change represents an extra challenge to livestock production systems. Rising temperatures and more frequent extreme weather events disrupt ecosystems, reduce feed availability, and intensify the spread of diseases, further endangering the sustainability of farming systems and the survival of small ruminants’ local breeds [9]. Numerous sheep and goat breeds have already disappeared, with many others currently at risk of extinction [10]. In this context, preserving animal diversity has become essential to ensure that future generations address emerging challenges and needs.

Italy is a country that is rich in history and has diverse environmental conditions and wide-ranging cultural traditions. It has helped preserve the small ruminant population, which includes over 80 distinct breeds of sheep and goats [11]. Italy is one of the largest European countries in terms of the number of sheep and goat herds, with an estimated population of about 5.4 million sheep and 1 million goats (Ministry of Health—National Veterinary Information System; https://www.vetinfo.it, accessed on 22 January 2025). The region of Apulia has the fifth most sheep and goat sector farms in the country, with about 3598 farms (Regional farmers association, A.R.A., data provided on January 2025) and 195,344 animals (Ministry of Health—National Veterinary Information System; https://www.vetinfo.it, accessed on 22 January 2025). The small ruminant farms in this region are located mainly in marginal areas characterized by strong traditional, agro-pastoral, and low-input farming practices well-adapted to the local environment. In this area, sheep and goat farming sustains rural communities, providing livelihoods and maintaining traditional practices. 

Understanding the characteristics of the sheep and goat local breeds is essential for developing effective strategies for their preservation and improvement, thereby supporting both animal biodiversity and sustainable agriculture. Indeed, autochthonous livestock breeds hold significant value as they preserve unique genetic diversity distinct from mainstream production breeds. This diversity could become crucial as genetic resources for domestic animals in response to future challenges like climate change and the increasing demand for food in the future [6,12].

In the last years, the European Union has effectively placed the issue of safeguarding the landscape and biodiversity among the objectives of the current agricultural policy, making it one of the pillars on which the New Green Deal is based. Biodiversity protection represents a challenge included in the Agenda ONU 2030 for Sustainable Development Goals (SDGs; Target 2.5). In particular, recent biodiversity policies emphasize the importance of adopting a whole-of-society approach to safeguarding biodiversity (EU Biodiversity Strategy for 2030) [13]. This strategy underscores the need for incorporating biodiversity considerations into decision-making processes across public and private sectors at all levels. Addressing biodiversity is challenging because the effectiveness of measures is context-specific [14], and it is further complicated by the multitude of elements linked to the specific ecosystem.

Targeted strategies have been developed to prevent the loss of small ruminant biodiversity. Phenotypic and genetic characterization of breeds represent a viable strategy to facilitate the creation of breeding plans for conservation purposes [15]. Beyond this, the production and promotion of traditional dairy products derived from autochthonous breeds of goats and sheep plays a direct role in biodiversity protection and maintenance. Indeed, these products support economic development within rural communities. Apulia boasts an ancient and high-quality dairy tradition, which has given rise to a variety of unique and renewed typical products from small ruminants including those recognized as Protected Designation of Origin (PDO) and Traditional agri-food products (TAP). Typical Apulia dairy products reflect the relationship between the land, the sea, and the complex historical and cultural influences of the various civilizations that have shaped this region. The connection between local breeds and their traditional products represents an opportunity to convert abandoned marginal areas into sites of cultural interest and food tourism. 

The main purpose of the present review was to provide insights about the autochthonous goat and sheep breeds of the Apulia region, highlighting their characteristics and significance, as well as their historical role and traditional productions, with a focus on the dairy products. A further goal is to propose strategies that can protect and promote biodiversity conservation of these endangered autochthonous breeds.

## 2. Role of Small Ruminants’ Autochthonous Breeds in the Culture Heritage of Apulia Region

The Italian territory is characterized by a rich environmental diversity, with habitats that span from alpine to a Mediterranean climate. Archeological data suggest that the early introduction of domestic small ruminants in the country occurred in south-eastern Italy at least ca. 8000 years B.P., likely as a consequence of maritime colonization events [16]. Apulia is one of Italy’s key agricultural regions, with its primary sector serving as a rich repository of significant historical and cultural heritage. The region exhibits a typical Mediterranean climate, characterized by a hot, dry summer and a mild, wet winter. Average annual temperature range between 14 °C and 18 °C (average summer temperature often exceed 30 °C) while winter temperature rarely falls below 5 °C. The region receives limited annual rainfall, averaged at 400–800 mm/year, mainly concentrated in the cooler months (autumn and winter) according to different measuring stations (Weather Data ARPA Puglia, http://www.webgis.arpa.puglia.it/meteo/index.php; accessed on 16 May 2025). Apulia’s topography features a wide range of altitudes, with a maximum height of 1152 meters (3780 ft) at Monte Cornacchia in the Daunia Mountains, and sea level along the coastlines (Apulia topographic map, https://en-gb.topographic-map.com/map-jnhgt/Apulia/?center=41.70409%2C15.61981&zoom=8; accessed on 16 May 2025). Apulia is covered by natural vegetation, including forests, grazing areas, and fallow land, totaling approximately 37,000 hectares [17]. The flora composition of the grazing areas (pasture sward) is predominantly represented by Poaceae (46%), Fabaceae (10%), Asteraceae (11%), Lamiaceae (4%), Liliaceae (8%), Caryophyllaceae (0.4%), Brassicaceae (1.6%), and Rubiaceae (2%). Additionally, a miscellaneous group of species belonging to various botanical families, representing about 17% of the total flora, was abundant in certain pasture areas of the Apulia region, including Boraginaceae, Dipsacaceae, Euphorbiaceae, Iridaceae, Plantaginaceae, Ranunculaceae, Scrophulariaceae, and Apiaceae, with variable levels of representation [18]. The variation of flora composition of the pastures depends on pedoclimatic, meteorological conditions, and the plants’ phenological stage, as well as the feeding behavior.

The Apulia’s geographical position and topography, from coastal plains to mountainous areas, provide distinct ecological niches where sheep and goats contribute significantly to the genetic richness of the Mediterranean livestock pool. For millennia, traditional pastoralism has been practiced in managing sheep and goat populations in the Apulia region, which involved low-intervention farming practices. Farmers and pastoralists selected animals for traits that suited their local environment, such as drought resistance, disease tolerance, and the ability to thrive on low-quality forage. Over time, this selective breeding, combined with the influence of natural selection, led to the emergence of local breeds adapted to different geographic and agro-climatic conditions and capable of utilizing marginal lands [11,19]. Autochthonous breeds are well adapted to local environmental and management conditions of the areas where they originated; therefore, they exhibit enhanced resilience to disease and reduced dependence on external inputs, such as commercial feed, veterinary services, and pharmaceutical drugs [20,21]. Pieragostini et al. [22] reported that the reduced veterinary costs in the Gentile di Puglia breed were related to the animals’ ability to be more resilient to climatic stress and endemic tick-borne parasites.

Selective grazing behavior of autochthonous breeds plays a crucial role in controlling weeds and invasive plant species, thereby preventing shrub encroachment and reducing the risk of wildfires [23]. This contributes to improved soil health and fertility, reducing the need for chemical inputs such as herbicides and fertilizers, which then supports more resilient agroecosystems. By maintaining open landscapes and fostering biodiversity, these autochthonous breeds play a pivotal role in agroecological management and the preservation of Apulia’s rural environment and ecosystem [24].

**Figure 1:**
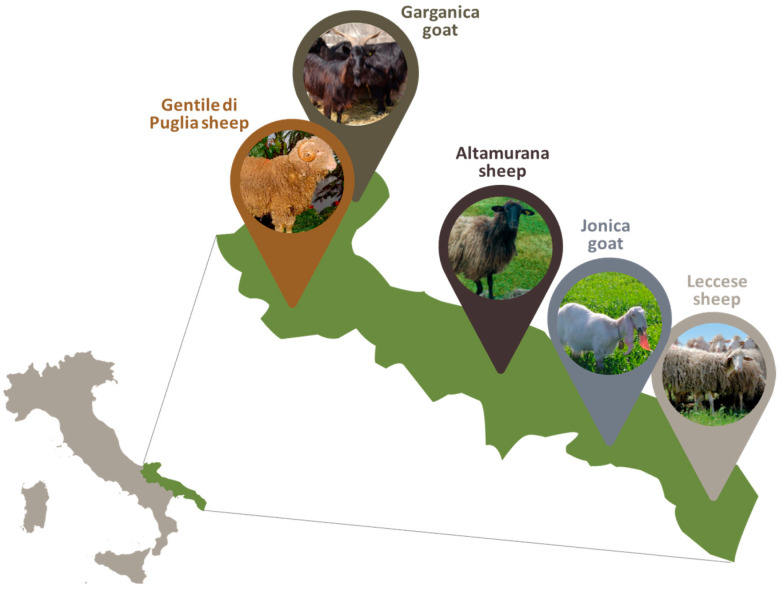
Distribution of the small ruminant autochthonous breeds in the Apulia region.

Apulia’s small ruminant biodiversity is enriched by different autochthonous sheep and goat breeds, including Gentile di Puglia, Altamurana, and Leccese sheep breeds and Garganica and Jonica goat breeds, which have a prevalent geographical localization in Apulia region (Figure 1).

These breeds constitute a valuable genetic heritage providing a high-quality milk essential for the production of the traditional dairy products [25]. 

The traditional agro-pastoral systems are still important activities in terms of employment and income, but also for ecosystem functioning and landscape management [26]. Recently, transhumance was inserted into the UNESCO list of the intangible cultural heritage of humanity, in recognition of traditional practices in parts of Italy, Austria, and Greece. The traditional farming practice of seasonal migration of livestock along herbal tracks connecting flatlands to uplands is practiced to provide better climate and feeding conditions for the herds throughout the year. In Apulia, transhumance has an ancient origin with a long-documented history, particularly involving small ruminants’ autochthonous breeds [27]. The transhumance routes covered more than two hundred kilometers, along the “tratturi” broad (also called “royal” paths), ancient trails measuring up to 100 meters in width. These pastoral roads connected the lowland grazing areas of the Apulia region with the mountain pastures of the Abruzzo Apennines, enabling the seasonal migration of flocks in search of better forage and milder climates. Effectively, through its connection to the use and management of pastures along the extensive network of sheep tracks, transhumance significantly contributed to the creation of a peculiar agricultural landscape and to the development of a rich mosaic of pastoral landscapes and their associated habitats in the Apulia region.

**Figure 2:**
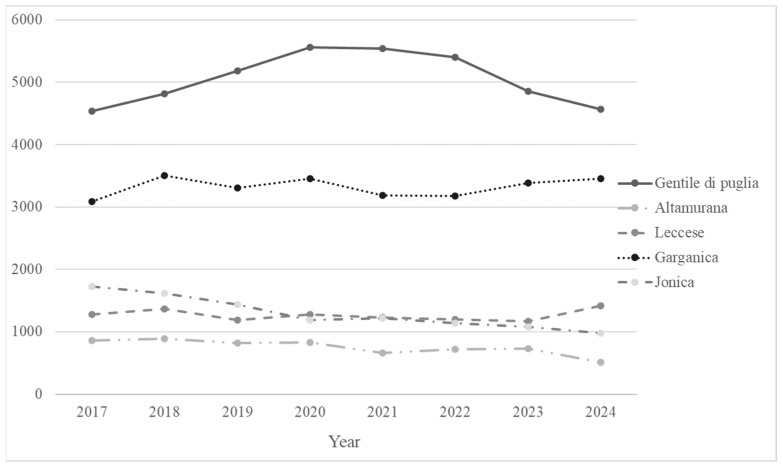
Autochthonous sheep and goat breeds population trends (2017–2024) in The Apulia Region. Source: National Zootechnical Register Statistics.

Population trends of the five principal autochthonous sheep and goat breeds in Apulia Region is reported in Figure 2.

## 3. Principal Small Ruminant Autochthonous Breeds of Apulia Region

### 3.1. Gentile di Puglia Sheep Breed

Gentile di Puglia, literally “Gentle Apulian” because of its fine wool, is a multipurpose sheep breed of millenary origin in Southern Italy, mainly in the area of Foggia named Tavoliere delle Puglie and Monti Dauni in the Apulian region. This breed was created by crossbreeding of Spanish Merino rams and local “Gentile” ewes. After the Second World War, the Gentile di Puglia was the dominant sheep breed in Southern Italy, with a population reaching almost 1 million animals [28]. Thereafter, this autochthonous breed has undergone a dramatic reduction, driven by many factors such as (i) the Gentile di Puglia breed’s low milk yield; (ii) decline of the wool industry, which faced increasing competition from plant-based and synthetic fibers; (iii) transformation of the production system from transhumance to stationary [29,30]. In order to sustain their livelihood, farmers crossbreed the Gentile di Puglia breed with more productive breeds, generating a new genetic type, the “Merinizzata Italiana”, which further contributed to the breed’s decline [31]. 

In 1983, the Gentile di Puglia breed population was estimated at 500,000 head. Nowadays, the last census conducted by the Italian national association of sheep and goat-breeders [32] reported a population of about 4500 herd book-registered animals (Figure 2), including 550 rams and 3900 ewes. This breed is classified as vulnerable by the Domestic Animal Diversity Information System (DAD-IS) of FAO (https://www.fao.org/dad-is/browse-by-country-and-species/en/; accessed on 19 May 2025). Historically, Gentile di Puglia sheep were reared under a transhumant pastoral system, characterized by seasonal migrations; from October to May, sheep grazed on the extensive pastures of the flatlands of Tavoliere delle Puglie, while during the summer months (June to September), they were moved to the mountainous regions of Molise and Abruzzo in search of more favorable climatic and nutritional conditions. For centuries, this breed has been a symbol of transhumant livestock management, closely linked to traditional rural infrastructure and cultural practices [33]. 

The Gentile di Puglia sheep, originally selected for fine wool production, demonstrates moderate milk yields ranging from 89 to 100 liters per lactation, a prolificacy rate of 120%, and good fertility under extensive rearing systems (90%; https://www.assonapa.it/public/schede-ovini/GENTILE-DI-PUGLIA.pdf; accessed on 16 May 2025). At present, the Gentile di Puglia sheep are currently raised for multipurpose production; (i) milk, due to its unique nutritional composition, which is rich in fat and proteins, is destined for production of traditional cheeses like Pecorino [34], and Scamorza [35]; (ii) meat, produced from lambs characterized by a high nutritional protein and fatty acids profile and slaughtered as light lambs to meet the consumer market demand [36]; (iii) excellent wool quality characterized by ultrafine and fine fiber classes and good fleece homogeneity [37]. In 2023, the Gentile di Puglia breed has gained the Slow Food Presidium designation for preserving a unique and cultural heritage (Slow Food Foundation for Biodiversity, www.fondazioneslowfood.com; consulted on 10 April 2025).

### 3.2. Altamurana Sheep Breed

The Altamurana sheep breed originated from Asian or Syrian breeds (Ovis aries asiatica); the breed is primarily raised in two areas of the Apulia region, which are the provinces of Bari and Foggia. The Altamurana sheep, named from the town of Altamura in the Murge area, is a medium-sized indigenous breed with long fleece wool and an elongated head, well-suited to harsh climates and high-altitude environments [38]. 

The Altamurana breed, known its remarkable rusticity and adaptation to arid environments, produces about 80 to 120 liters of milk per lactation, which lasts for approximately 120 days (https://www.assonapa.it/public/schede-ovini/ALTAMURANA.pdf; accessed on 16 May 2025). 

This breed is characterized by high fertility rates (>90%) and is particularly resistant to piroplasmosis, a disease that can quickly be fatal to infected sheep. Despite its strong disease resistance, the breed has suffered a dramatic population decline over the past three decades. In 1984, the Altamurana breed population was estimated at 190,000 head, but in 2012, this number collapsed to just 230 head, classifying the breed as endangered, maintained by DAD-IS of FAO (https://www.fao.org/dad-is/browse-by-country-and-species/en/; accessed on 19 May 2025). The Altamurana is one of the autochthonous Italian sheep breeds, such as Leccese, Sarda, Comisana, Pinrizita, Delle Langhe, Massese, and Valle del Belice, for which a genealogical herdbook is kept by ASSONAPA. Currently, about 550 animals are registered in the herd book, with 93% located in Apulia (508 heads, Figure 2) and 6% in the Basilicata region (https://www.vetinfo.it/; accessed on 31 December 2024). 

Altamurana sheep is a multipurpose breed reared for cheese and meat production. Altamurana milk is processed to obtain cheese with nutritional characteristics and sensorial properties distinguishable from non-native breeds in terms of fatty acid profile, VOC compounds, and sensory properties [39]. Regarding meat production from Altamurana breeds, light lambs are usually consumed according to the traditional gastronomic preparation. However, a comparison between Altamurana lambs slaughtered at 40 and 75 days of age evidenced different nutritional and organoleptic features, showing a relationship between fatty acids profile and sensory attributes [40]. These findings could help meet consumers’ expectations and contribute to valorize meat production from Altamurana breeds. 

### 3.3. Leccese Sheep Breed

The Leccese breed, also called Moscia Leccese, traces its lineage to the ancient Zackel sheep of Asia (*Ovis aries asiatica*), reflecting a rich heritage rooted in centuries of pastoral tradition. With their unique, loosely hanging wool coat, they embody a rustic elegance that echoes their nomadic ancestry [41]. Leccese sheep are a medium-sized breed primarily raised in Salento, the southern part of Apulia, characterized by a semi-arid climate with long, dry and hot summers and mild winters. The Leccese sheep stand out with their rosy skin and white coat, adorned by bold black spots on their breastbone. Their short-haired black muzzle and limbs lend them a rugged elegance that reflects their resilience and adaptability in the rocky and sparse pastures of the Apulian uplands [42]. 

In 1984, the Leccese breed population was estimated at 240,000 head. In the last census, the Italian National Livestock Registry (https://www.vetinfo.it/; accessed on 22 January 2025) reported that the Leccese breed counted 1575 head, mainly reared in farms located in the Apulia Region (90%, 1414 heads; Figure 2). At present, the Leccese breed is classified as endangered, maintained by DAD-IS of FAO (https://www.fao.org/dad-is/browse-by-country-and-species/en/; accessed on 19 May 2025). The Leccese breed is widely recognized for its tolerance for high temperatures and resistance to photoperiodic effects, high milk yield (150 liters per lactation), and fertility rate of 90% with a high prolificacy of 125%. Leccese sheep are known for their (i) white wool, traditionally used in textile production; (ii) high-quality protein-rich milk, which is ideal for making ricotta and Pecorino cheeses; and (iii) lamb meat production. A survey on the genetic polymorphism of the β-lactoglobulin gene in Leccese sheep showed that protein alleles had an effect on milk composition in terms of fat and whey protein content [43]. Regarding meat quality, intramuscular fat of longissimus lumborum from Leccese lambs was characterized by a fatty acids profile that is more favorable to meeting nutritional requirements for human health at both 45 and 60 days of slaughtering; only CLA content was improved by the slaughter age at 60 days [44]. A further study showed that consumers found the meat of Lecese lambs to be more acceptable when it was sourced from lambs fed by grass-fed ewes than those reared in stalls on concentrate [45].

### 3.4. Garganica Goat Breed

Garganica is an ancient autochthonous breed that originates in the Gargano promontory of the Apulia region, obtained by crossing the autochthonous goat population with Western European goats. Garganica goats are primarily raised in the Gargano promontory as well as in other regions of Southern Italy (Sicilia, Basilicata, and Calabria). Garganica are medium-sized animals with glossy black coats that may display reddish hues and long, twisted horns in both sexes. The Garganica goat is usually raised in medium to large herds under wild or semi-wild conditions; they have exceptional adaptability to harsh environments and the ability to utilize poor pastures. The Garganica goat breed is classified as one of Italy’s vulnerable breeds (FAO, https://www.fao.org/dad-is/browse-by-country-and-species/en/ accessed, 19 may 2025). At present, about 6400 Garganica goats are reported in the Italian National Livestock Registry (https://www.vetinfo.sanita.it; accessed on 22 January 2025); of these, 54% are reared in Apulia (3451 heads; Figure 2) and 38% in the Basilicata region. Garganica goats are characterized by a strong resilience and adaptability, with a fertility of 95% and a twinning rate of 20%.

The Garganica goat is primarily recognized as a dairy breed, reared in mountainous and marginal areas, producing between 180 and 250 liters of milk per lactation, with lactation durations up to 180–200 days. Garganica goat milk is primarily used in the production of traditional dairy products as Cacioricotta cheese [46]. Research explored the role of Garganica goat milk in human health, especially in the prevention of childhood pathologies such as cow milk allergy, epilepsy, and obesity. The key factor for this benefit is the high genetic polymorphisms of the milk protein, which is conserved in the less selected goat breeds, such as Garganica breed. The variability of goat casein loci in this breed led to different level of total casein in milk, particularly the presence of null alleles, which involve the absence of the corresponding protein in the milk proteome. This could be exploited to differentiate the goat population on the basis of milk utilization: animals with strong alleles at CSN1S1 and CSN2 could be destined to produce milk for cheesemaking, and animals with weak or null alleles could be exploited in breeding programs aimed at producing milk with hypoallergenic properties [47].

The main typology of goat meat appreciated by the consumer is the kid named “capretto”, which is characterized by its rosy color. It has a delicate, wild taste due to kids suckling milk from goats that graze on spontaneous pastures and scrubs typical of the Mediterranean area. This spontaneous natural feeding is able to improve milk and meat nutritional quality with beneficial function on human health. A study on dietary supplementation of Garganica kids with dried oregano inflorescences reported lower muscle fat oxidation and a better meat flavor that was highly appreciated by the consumer [48].

### 3.5. Jonica Goat Breed

The Jonica goat breed originated in the second half of the XX century from an autochthonous population of goats in the Ionian Arc area that has been repeatedly crossbred with the Maltese breed. The Jonica goat is characterized by a distinctive trait of the Maltese goat breed, its long and wide ears, which hang down on both males and females. The Jonica goat is a medium to large-sized breed with a small, light head and long slender limbs. Its white fleece may occasionally have a slight pink hue, with tawny speckles or spots on the head and neck. Males also have a tuft of matted hair on the top of their heads. Native to the Salento area of the Apulia region, the Jonica goat has grazed for centuries in the historic Terra d’ Otranto, which includes the provinces of Brindisi, Lecce, and Taranto. This area of the Apulia region is characterized by lands parched by the summer heat, with shepherds guiding their flocks through the countryside, navigating olive groves and dry-stone walls in search of sustenance. Indeed, the Jonica goat is highly adapted to grazing on arid soils and can utilize food resources that would otherwise go unused [44]. At present, there are about 1300 Jonica goats registered in Italy (Italian National Livestock Registry; https://www.vetinfo.it/; accessed on 22 January 2025); of these, 76% are reared in the Apulia farms (Figure 2), 11% in Sicily, and 6% in the Basilicata region. This breed is classified as critical, maintained by FAO (https://www.fao.org/dad-is/browse-by-country-and-species/en/; accessed on 19 May 2025). The Jonica goat is characterized by high prolificacy of about 217%, high fertility (97%), and rates close to 210%.

The Jonica goat is recognized as a dairy breed, characterized by high milk yields that often reach 220 to 340 liters per lactation under semi-intensive systems. However, it is important to underline that Jonica goat milk is mainly used to produce fresh ricotta, ricotta forte, and ricotta marzotica in the spring, while Cacioricotta cheese is produced during the summer months [49].

## 4. Developing Local and Traditional Premium Products with Distinctive Qualities

Local and traditional agri-food products have seen increasing interest from Italian consumers in recent years, who expressed a marked preference for buying products produced predominantly in Italy (also defined as “made in Italy”; 74.1%), whose origin is close to the point of purchase (“km 0” products; 59.3%), and seasonal (80.4%) [50]. Despite these trends, the definitions of “local product” and/or “traditional product” remain unclear, lacking universal classification criteria. Generally, a product is considered local if the marketing area aligns with the production area, although there is no comprehensively accepted definition of which constitutes a local area. Traditional products are defined as “goods frequently consumed or associated with specific celebrations or seasons, traditionally passed down through generations, produced in accordance with culinary heritage, with minimal processing or handling, renowned for their unique sensory characteristics, and closely connected to a specific local area, region, or country” [51].

In recent years, more attention has been paid to sheep and goat milk and high-value dairy products, which are often processed in small local dairies. Apulia is widely regarded as a leading Italian region with the oldest cheesemaking traditions that use sheep and goat milk. This heritage is characterized by the use of traditional and distinctive production methods passed down through generations and the production of small-scale batches that prioritize artisanal practices with minimal support from mechanization. Typical and local products, which embody identity, heritage, history, tradition, and quality, are deeply interrelated with the rural landscape that recalls the scents of the natural pastures where the animals graze. They represent cultural significance, ancient traditions, and collective knowledge, forming a valuable heritage including both cultural and biodiversity. Indeed, despite being geographically confined, these niche products serve as unique symbols of their regions of origin by giving a cultural identity, and they have the potential to drive social and economic development within their regions by supporting niche markets and agrotourism. 

**Figure 3:**
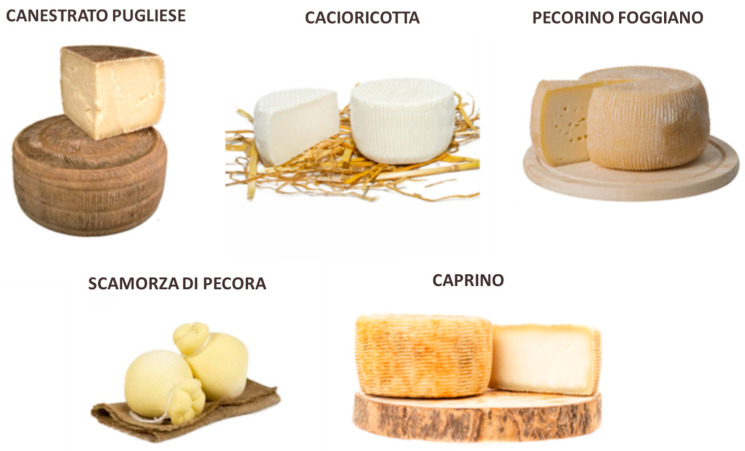
Apulian cheeses made from autochthonous sheep and goats.

Protected Designation of Origin (PDO), Protected Geographical Indication (PGI), and Traditional Agri-food Products (TAP) are specific European designations of agri-food quality that emphasize the safeguards and protection of agricultural and food products. These designations are explicitly meant to provide consumers with detailed information about products “obtained using processing, storage and maturation methods over time consolidated, consistent throughout the territory concerned, according to traditional practices”. The distinctive traits of sheep and goat dairy products from the Apulia region result from several factors, such as the type of milk, protocol production, ripening time, and the use of raw milk or heat-treated milk. Currently, one PDO and four TAP cheeses (Figure 3) from sheep and goats are recognized in the Apulia region (https://www.masaf.gov.it/flex/cm/pages/ServeBLOB.php/L/IT/IDPagina/21121; accessed on 22 January 2025), which are outlined in the following section.

### 4.1. Canestrato Pugliese

Canestrato Pugliese is a traditional Apulian uncooked hard cheese, manufactured by using milk from autochthonous sheep breeds that has had a Protected Designation of Origin (PDO; European quality label) status since 1996 [52]. Since sheep lactation primarily occurs between February and May, the Apulian lowlands bustle with shepherds producing cheese, the most notable of which is a hard type well-suited for long-term storage. The name of this cheese, Canestrato, originates from the rush basket “canestro” in which the curd is ripened. These baskets, known also as “fiscelle”, are one of the most traditional artisanal products of Apulia. The production of Canestrato cheese follows the production process derived from the Apulian tradition and uses sheep milk that is raw or thermally treated at 36–40 °C (Table 1). Natural whey cultures, primarily consisting of thermophilic lactic acid bacteria, may be included, while coagulation is induced using liquid or lamb rennet paste. Coagulation occurs in 10–15 minutes, and after clotting time, the coagulum is cut into 2–5 mm pieces and heated to 40–42 °C. The cheeses are dry-salted and ripened for a period ranging from 2 to 10 months in the “canestro”, then turned and rubbed with a mixture of oil and vinegar. In the industrial production of Canestrato, ripening in “canestro” lasts only a few days. Over time, molds from the surrounding environment often develop on the surface, but these are removed by brushing after several months. The Canestrato cheeses are cylindrical, measuring between 10 and 14 cm in height and 25 to 34 cm in diameter, with a weight ranging from 7 to 14 kg. 

The rind ranges in color from pale yellow to brown, while the interior is dense with small holes. The flavor is unique and tends to a moderate piquancy. Measuring sessions allowed the panel to judge Canestrato Pugliese made from pasteurized and raw milk according to a set of attributes, including odor, flavor, and taste. Attributes for odor were butter, ewes’ milk, stable, mushroom, cream, and rennet. Attributes for flavor were butter, ewes’ milk, stable, mushroom, and cream, and finally, the attributes for taste were salty and pungent. Milk pasteurization was a fundamental source of variation; Canestrato Pugliese made from raw milk was characterized by higher notes of ewes’ milk, stable, and mushroom odor and aroma, and higher intensities of pungent and salty tastes [53]. The chemical composition of Canestrato Pugliese cheese is reported in Table 2. 

Albenzio et al. [25] examined the impact of milk pasteurization and of heating the curd in hot whey (80 °C for 30 s) on proteolysis and lipolysis; both treatments led to a reduction in lactic acid bacteria counts and slower proteolytic and lipolytic processes compared to that produced from raw milk. Furthermore, a complex microbial ecology was characterized in the Canestrato pugliese obtained by using raw milk [58]. Sensory properties of Canestrato Pugliese cheese made from raw and pasteurized milk evidenced significant differences in the volatile fraction composition exhibiting a threefold higher level of free fatty acids [59]. 

### 4.2. Cacioricotta Goat Cheese

Cacioricotta cheese is a traditional dairy product from the Apulia region produced from whole goat milk, sometimes blended with ewes’ and/or cows’ milk, following traditional artisanal cheese-making methods deeply rooted in local farming practices. Indeed, its production typically occurs at a non-industrial scale linked to a seasonal transhumance period, reflecting its strong connection to traditional pastoralism. Cacioricotta goat cheese is included in the Sixteenth Revised Regional and National List of “Traditional agri-food products” [60]. 

Cacioricotta cheese is produced from raw milk which undergoes heating at about 90–95 °C; this step differentiates it from many other cheeses as it retains casein and whey proteins in the curd. The heated milk is than cooled, and liquid veal rennet is added. As a result, the curd has a reduced tendency for syneresis due to the incorporation of whey proteins into the paracaseinate network. After curdling, the cheese is either consumed fresh, as a soft cheese, after less than two days of ripening (extra-rapid ripening), or after 15 days (semi-soft cheese) or more of ripening (2–3 months maximum) to develop a firmer texture and intense flavor ideal for grating. 

Cacioricotta goat cheese has been extensively characterized from both nutritional and organoleptic perspectives [46,56,57,61]. It was reported that the high-temperature treatment not only improved the rennet clotting time but also enhanced the cheese’s nutritional profile, leading to a higher taurine and protein content than cheeses made from unheated milk; taurine is a sulfonated amino acid with important physiological roles [62,63]. The chemical composition of Cacioricotta is reported in Table 2. To improve the yield and organoleptic characteristics of Cacioricotta cheese, Caponio et al. [56] investigated the impact of reducing the amount of rennet (from 100 to 40 mL/q) and adding a mesophilic starter mixed with the conventional thermophilic starter in the cheese-making process. Data revealed that Cacioricotta cheese produced with the modified process showed higher moisture, proteins, and fat contents, as well as a lower concentration of short- and medium-chain fatty acids which produced aromatic compounds, thus improving its organoleptic characteristics. Celano et al. [57] reported that Cacioricotta has higher carbohydrates and sodium chloride and low peptidase activity. Gas chromatography coupled with mass spectrometry (GC-MS) and olfactometry allowed for the quantification and identification of the characteristics volatile compounds (VOC); among these, 1-Butanol,3-methyl and phenylethyl alcohol were identified as the most abundant in Cacioricotta cheese. 

Goat milk from purebred Garganica goats was used to make Cacioricotta cheese during the spring season, and the chemical, enzymatic, and proteolytic characteristics were investigated [46]. The results of that study showed that the quality of Garganica raw milk impacted the nutritional characteristics and proteolytic patterns of Cacioricotta cheese in terms of somatic cell count and the cheese-making procedures. The authors emphasized that the sanitization of housing and milking procedures are critical for optimizing the nutritional and hygienic characteristics of Garganica goat milk, and consequently, of the Cacioricotta Garganica goat cheese.

### 4.3. Pecorino Foggiano Ewe Cheese

Pecorino Foggiano is one of the oldest typical TAP cheeses of Capitanata (province of Foggia, Apulia region), strictly related to the seasonal transhumance from the Abruzzo mountains to the Tavoliere delle Puglie (flatlands extending from the Gargano promontory to the Dauno Apennine of the Apulia region) (Table 1). During the Aragonese period, two distinct varieties of Pecorino were produced, called “Pecorino Foggiano”, characterized by a large size and piquant flavor, and “Pecorino Dauno” characterized by a shorter ripening period. Both kinds of cheese were produced from the milk of local merinized sheep breeds, such as the Gentile di Puglia, following traditional and artisanal methods. To produce Pecorino Foggiano, sheep milk is heated to 37–43°C and coagulated by lamb rennet paste. The clotting time is 12–14 min. The curd is cut using a special wooden stick, either an m’natur, which is smooth and spoon-shaped, or a spinu, which is smooth and spiked. The resulting curd is placed into rush baskets and pressed by hand. Then, cheeses are dry salted for 3–7 days and ripened on wooden boards in cool and airy rooms, from 2 months for fresh Pecorino and 6–8 months for mature cheese Pecorino. The short-ripened type (2 months of ripening) has a soft and light yellow paste with slight eye, whereas the long- ripened type (up to one year) has a harder texture, a more pronounced color, and very intense piquant flavor [64]. During ripening, the cheeses are periodically greased with extra virgin olive oil and vinegar. The chemical composition of Pecorino Foggiano cheese is reported in Table 2. Celano et al. [57] investigated the microbiota, enzymatic activities, degree of proteolysis, and concentrations of the main volatile organic compounds (VOC), showing that some VOC are distinctive traits of Pecorino, such as it being rich in carboxylic acids, ketones, and esters, which are related to lipolysis.

Traditional Pecorino cheese has evolved by incorporating rennet containing free or encapsulated probiotics enhancing the nutritional value of the cheese without altering the traditional cheese-making method [65]. This approach preserves the Pecorino cheese’s traditional identity while addressing the evolving consumer demand for health-conscious products. Indeed, the encapsulation of probiotic *Lactobacillus acidophilus* and *Bifidobacterium lactis* (BB-12) and *longum* (BB-46) into lamb rennet paste influenced the nutritional, proteolytic, and rheological characteristics of Pecorino cheese. From a nutritional point of view, the use of probiotics contributes to ameliorating the free fatty acid (FFA) profile and the content of health beneficial compounds like conjugated linoleic acid (CLA) in the Pecorino cheese [34,65]. Additionally, probiotic strains appear to modulate the enzymatic activity, affecting peptide degradation, amino acid release, and lipolysis, improving the texture and flavor sensory characteristics of Pecorino Foggiano cheese obtained from Gentile di Puglia ewe milk [34].

### 4.4. Scamorza Pasta Filata-Type Cheese from Ewe Milk

Pasta filata-type cheese is a traditional cheese produced in Italy that is usually made from cow and buffalo milk. In Southern Italy, Scamorza cheese is a typical pasta filata cheese characterized by short or medium ripening time. In the Apulia region, Scamorza cheese is also produced by using ewe milk from pasture-raised autochthonous breeds (Gentile di Puglia, Leccese, and Altamurana) and undergoes a short ripening period (7–10 days) [49]. The word Scamorza means to behead, and this reflects the cheese’s characteristic pear shape, which features a short head-shaped neck on a rounded body. The cheese is traditionally handmade. The principal phases of Scamorza cheese production are the acid development, rennet catalyzed coagulation, heating at 80 °C, and stretching of the curd mass. The stretching phase is allowed by the proper acidification of the curd, which is usually cut into cubes or stripes and matured until it reaches pH 4.9. This pH value confers the optimum stretchability to the sheep milk curd. The process of heating and stretching results in the visible alignment of protein fibers within the curd, which entraps coalesced fat and moisture, giving the cheese a glossy, white appearance. The thermal treatment also significantly impacts the cheese’s microbiological, biochemical, physicochemical, and functional properties, which in turn influence its sensory characteristics. After a short ripening time, its consistency becomes firmer, and it loses some elasticity. The chemical composition of Scamorza cheese from ewe milk is reported in Table 2. The combination of traditional methods with innovative approaches, such as the incorporation of probiotics in the Scamorza cheese manufactured with ewe autochthonous milk, offers the possibility to develop novel products with different sensory characteristics, potentially increasing the appeal of traditional products in the market and among different types of consumers. Indeed, Albenzio et al. [35] have examined the addition of probiotic strains, including *Lactobacillus acidophilus* and a combination of *Bifidobacterium longum* and *Bifidobacterium lactis*, into the Scamorza ewe cheese matrix during production. That study showed that the addition of probiotic bacteria influenced the Scamorza ripening process, leading to greater proteolysis and lipolysis and leading to a more complex soluble peptide and FAA profile as well as increased vaccenic and oleic acids and CLA content. Furthermore, the use of probiotics in Scamorza cheese affected the cheese’s texture and sensorial profile in terms of structural uniformity, friability, creaminess, and graininess. The definition of a specific quantitative vocabulary for Scamorza cheese sensory analysis and a specific reference frame for assessor training has been developed; this framework is useful not only to evaluate the sensory profile of traditional Scamorza cheese but also to monitor the quality of innovative typology of ewe milk cheese.

### 4.5. Caprino Goat Cheese

Caprino is a traditional long-ripened Apulian cheese made exclusively with raw goat milk, recognized as a TAP product in 2001. It has a flattened cylindrical shape, a diameter ranging from 10 to 23 cm, and a heel height of 5–7 cm. In the fresh cheese, its surface is wrinkled and white. It thickens and becomes firmer during ripening, developing a straw-yellow color. This cheese is characterized by rich flavor and distinctive aroma. For Caprino cheese production, raw goat milk is filtered directly into tin-plated copper tanks using fine-weave cotton cloths or a fine mesh strainer. The milk is then heated to 36–37 °C before adding liquid rennet from calf, kid, or lamb. Coagulation occurs within 15–45 minutes, followed by a 15-minute firming period before the curd is broken into small pieces using a wooden pin called a Ruotolo. A key step of the protocol production is cooking the curd-whey mixture at about 45 °C; after a brief resting period, the curd is manually pressed to the bottom of the tank, and then placed into rush, called “fische”, or plastic molds. Scalding of the curd in hot whey, called “scotta”, is performed at 80–85 °C for few seconds with the aim of controlling hygienic quality and improving whey drainage. Subsequently, molds are positioned on sloping wooden shelves, with manual pressing for 30–40 minutes to facilitate whey expulsion. Dry salting is performed by sprinkling coarse salt on the upper surface, and then on the lower surface the following day. After 2–3 days, the cheeses are removed from the molds and transferred to cool, well-ventilated environments for 6–8 months of ripening time.

The nutritional characteristics of Caprino cheese are reported in Table 2. A study on characterization and valorization of Caprino goat cheese found that it had high peptidase activities due to the use of natural whey starter cultures which add microbial biomass leading to a release of peptides and free amino acids from the casein matrix. Peptidase activities also have an impact on the sensory profile of the Caprino cheese, which is characterized by pyrazine derivatives that confer a distinctive roast nut aroma to the cheese. Caprino cheese is also rich in aldehydes, some medium chain fatty acids, and their derived ethyl esters [57].

## 5. Ecosystem Services Provided by Autochthonous Sheep and Goat Breeds of Apulia Region

The concept of Ecosystem Services (ESs), introduced by the Millennium Ecosystem Assessment in 2005, emphasizes the vital link between ecosystems, including agroecosystems, and human well-being. Ecosystem Services refer to different benefits that humans receive from nature, which can be classified as both direct and indirect [66]. These benefits may be economic or embedded in socio-cultural values, including benefits that contribute to human identity, emotional well-being, and societal cohesion [67]. By integrating ecological, economic, and socio-cultural dimensions, the ES framework provides a more comprehensive understanding of how ecosystems support the foundations of human life. In the agroecosystems, this concept is especially relevant, as these systems not only produce food but also sustain biodiversity, maintain soil health, and offer cultural heritage value [68]. 

Ecosystem Services are formally classified into four categories [69]; (i) Provisioning services that provide tangible or energy related outputs like food, water, fuel, and timber; (ii) Regulating services that include natural processes such as climate regulation, flood prevention, waste treatment, and water purification; (iii) Supporting services that include essential ecological functions like soil formation, photosynthesis, and nutrient cycling that sustain other services; (iv) Cultural services that are related to recreational, aesthetic, educational, and spiritual benefits from ecosystems [70].

Autochthonous Apulia sheep and goat breeds play a pivotal role in maintaining and enhancing these services, thus contributing not only to agricultural productivity but also to ecosystem resilience and rural socio-cultural activities. In the following section, the main aspects of different types of services provided by Apulia small ruminants autochthonous breeds are outlined (Table 3).

### 5.1. Provisioning Services

Autochthonous Apulia sheep and goat breeds provide high-quality products including milk, meat, and wool, which often possess unique nutritional and sensory properties that are shaped by the local environment and traditional management practices. As provisioning Ess, the Gentile di Puglia sheep provide wool with high quality characteristics (fiber is highly curled, less than 6 cm long, and has a diameter less than 19 μm). Wool production supports artisanal textile industries, preserving cultural heritage and traditional craftsmanship [71]. In addition to wool, the Gentile di Puglia breed also provides nutrient-rich milk that is rich in fat and proteins and is used for the production of traditional cheese as Canestrato, Cacioricotta, Pecorino, and Scamorza. Altamurana and Leccese breeds are also renowned for their high-quality milk, which is similarly used in the production of several traditional Apulian cheeses (Canestrato, Cacioricotta, and Scamorza). Milk from autochthonous goat breeds was also used for the production of different TAP cheeses, like Caprino and Cacioricotta cheeses; these cheeses are highly valued both locally and internationally for their distinct flavors and nutritional profiles. The autochthonous sheep and goat breeds of Apulia also provide meat with excellent nutritional characteristics in terms of more favorable fatty acid composition and less saturated fatty acids (SFA) content. These products not only contribute to food security but also offer economic opportunities through niche marketing, thereby supporting rural livelihoods and promoting agro-biodiversity conservation. 

### 5.2. Supporting Services

Autochthonous Apulia sheep and goat breeds play a key role in maintaining a high level of biodiversity. The extensive rearing system applied for the sheep and goat autochthonous breeds promotes the preservation of natural landscapes, fostering diverse plant communities adapted to the Mediterranean pedoclimatic context. Through selective grazing, these animals prevent the dominance of invasive plant species, thereby preserving species coexistence and habitat heterogeneity, which are essential for long-term ecosystem stability. 

### 5.3. Regulating Services

Sheep and goat autochthonous breeds perform crucial roles in vegetation management, which directly impacts fire regimes, soil erosion, and landscape dynamics. Their grazing behavior reduces the accumulation of combustible biomass in shrublands and forest edges, effectively lowering wildfire risk in a region increasingly vulnerable to climate change-induced droughts and heatwaves. Additionally, the physical impact of their hooves on soil promotes aeration and organic matter incorporation, enhancing soil structure and water infiltration. This regulation of ecological processes is fundamental to maintaining resilient agroecosystems under current and future climatic challenges. 

### 5.4. Cultural Services

The presence of autochthonous sheep and goats in the Apulia region represents an important element of the region’s cultural landscape and collective memory. These sheep and goat breeds symbolize centuries-old pastoral traditions and also serve as living reservoirs of traditional knowledge, such as transhumance, which is still practiced in the breeding of small ruminants [27]. Their presence contributes to the preservation of cultural identity and social cohesion through their association with traditional festivals, artisanal food production, and rural tourism. Additionally, the conservation of these genetic resources supports inter-generational transmission of knowledge related to sustainable land use, and biodiversity conservation. Thus, maintaining native breeds plays a dual role in both cultural heritage conservation and ecological sustainability.

## 6. Conclusions

This review presented the principal small ruminant autochthons breeds reared in Apulia region as Gentile di Puglia, Altamurana, and Leccese sheep breeds and Garganica and Jonica goat breeds. It was outlined that the strict bound among sheep and goat farming, ecosystem, and cultural heritage in the Apulia region represents an approach to safeguard the landscape and animal biodiversity according to the EU Biodiversity Strategy for 2030. Based on the consulted scientific literature, dairy and meat products from the mentioned breeds are of high quality, especially the well-recognized PDO and TAP cheeses, but the literature also suggested a need for product and production process innovation, starting from the traditional and local products. 

The valorization of autochthonous small ruminant breeds is founded on genetic heritage, ecosystem functioning, and landscape management, as well as the improvement of healthfulness and functional properties of their production.

## Figures and Tables

**Table 1:** Main characteristics and parameters used during manufacture of traditional Apulian cheeses.

Cheese Denomina-tion	Sheep/Goat Milk	Designation	Milk Thermal Treatment	Type of Starter Culture	Type of Rennet	Salting	Ripening	Diameter and Thichness	Shape and Weight
Canestrato Pugliese	Gentile di Puglia, Altamurana and Leccese	PDO (1996)	None	None, or natural culture in whey or milk	Calf, powered or liquid	Dry for 4–6 days	2–10 months	25–34 cm diameter; 10–14 cm high	Cilindrical shape; 7–14 kg
Cacioricotta	Gentile di Puglia, Altamurana, Leccese sheep; Garganica and Jonica goats	TAP (2001)	Pasteourisation	None	Calf, liquid	Dry for 1 day	15 days extra-rapid ripening; 2–3 months long ripening	10–20 cm diameter; 7–13 cm high	Cilindrical shape; 0.4–0.6 kg
Pecorino Foggiano	Gentile di Puglia	TAP (2001)	Thermisation	Commercial	Lamb and Calf, liquid	Dry for 3–7 days	2 months (short ripening); 6–8 months (long ripening)	15–34 cm diameter; 6–12 cm high	Cilindrical shape; 2–7 kg
Scamorza di Pecora	Gentile di Puglia, Altamurana and Leccese	TAP (2001)	Thermisation	None	Calf, liquid	Brine for 2 hours	7–10 days	10–15 cm diameter; 10–12 cm high	Pear shape; 150–400 g
Caprino	Garganica and Jonica goats	TAP (2001)	Thermisation	Commecial	Lamb and Calf, liquid	Dry for 2–3 days	6–8 months	10–23 cm diameter; 5–7 cm high	Cilindrical shape; 2–3 kg

**Table 2:** Chemical composition of the traditional Apulian cheeses.

	pH	Aw ^a^	Protein (%)	Fat (%)	Carbohydrates (%)	Moisture (%)	References
**Canestrato Pugliese**	5.0–5.2	0.87	26.5	30	0.2	36.5–39.5	[25,39,54,55]
**Cacioricotta**	5.7–6.3	0.95	22–32	25–32	2.3–4.2	31–45	[46,49,56,57]
**Pecorino Foggiano**	5.0–5.2	0.87	20–25	28–32	0.2	34–42	[57]
**Scamorza di Pecora**	5.0–5.3	–	24–32	25–26	0.4	40–46	[35,49]
**Caprino**	5.2	0.88	26–28	23–29	0.2–0.7	34–39	[49,57]

^a^ water activity.

**Table 3:** Provisioning, supporting, regulating and cultural ECS services provided by autochthonous sheep and goat breeds of Apulian region.

Autochthonous Sheep/Goat Breed	Provisioning Services	Supporting Services	Regulating Services	Cultural Services
**Gentile di** **Puglia**	Wool; Milk for traditional cheese productions (Canestrato pugliese, Cacioricotta, Pecorino Foggiano, Scamorza di Pecora); Lamb meat	Conservation of local/autochthonous genetic resources; Resilience to local parasites; maintenance of high level of biodiversity (number of plant species present in pasture); preservation of natural landscape.	Landscape management; prevention of shrub encroachment; Fire risk reduction through grazing; maintenance of pasture dynamics.	Preservation of transhumance traditions; Identity linked to traditional wool and milk processing; Agrotourism and educational programs helping tourists and students connect with rural life.
**Altamurana**	Milk for traditional cheese production (e.g., Canestrato pugliese, Cacioricotta, Scamorza di Pecora), Lamb meat			Strengthening of rural heritage; promotion of agrotourism activity.
**Leccese**	Wool; Milk for traditional cheese production (e.g., Canestrato pugliese, Cacioricotta, Scamorza di Pecora), Lamb meat			Cultural identity of Salento linked to historic pastoral festivals.
**Garganica**	Milk for traditional cheese production (Caprino and Cacioricotta)	Conservation of local/autochthonous genetic resources; Resilience to local parasites; maintenance of high level of biodiversity (number of plant species present in pasture); preservation of natural landscape.	Soil erosion control in grazing systems; Prevention of forest fires in marginal areas; promotion of natural ecological cycles.	Symbol of mountain culture in the Gargano area linked to artisanal cheese making; Cultural transmission on history, traditions, and identity of the local communities; Link to traditional dairy products and rituals.
**Jonica**	Milk for traditional cheese production (Caprino, Cacioricotta, Ricotta forte, Ricotta Marzotica)			Educational farms and local biodiversity valorization; food festivals or “sagre” feature, goat cheese tastings, celebrating regional identity.

## Data Availability

The raw data supporting the conclusions of this review will be made available by the authors on request.

## References

[1] Nardone A., Zervas G., Ronchi B. (2004). Sustainability of small ruminant organic systems of production. Livest. Prod. Sci..

[2] Taberlet P., Coissac E., Pansu J., Pompanon F. (2011). Conservation genetics of cattle, sheep, and goats. Comptes Rendus. Biologies.

[3] Larson G., Fuller D.Q. (2014). The evolution of animal domestication. Annu. Rev. Ecol. Evol. Syst..

[4] MacHugh D.E., Larson G., Orlando L. (2017). Taming the past: Ancient DNA and the study of animal domestication. Annu. Rev. Anim. Biosci..

[5] Wykes D.L. (2004). Robert Bakewell (1725–1795) of Dishley: Farmer and livestock improver. Agric. Hist. Rev..

[6] Taberlet P., Valentini A., Rezaei H.R., Naderi S., Pompanon F., Negrini R., Ajmone-Marsan P. (2008). Are cattle, sheep, and goats endangered species?. Molec. Ecol..

[7] Kijas J.W., Townley D., Dalrymple B.P., Heaton M.P., Maddox J.F., McGrath A. (2009). International Sheep Genomics Consortium. A genome wide survey of SNP variation reveals the genetic structure of sheep breeds. PLoS ONE.

[8] Kijas J.W., Lenstra J.A., Hayes B., Boitard S., Porto Neto L.R., San Cristobal M. (2012). International Sheep Genomics Consortium. Genome-wide analysis of the world’s sheep breeds reveals high levels of historic mixture and strong recent selection. PLoS Biol..

[9] FAO (2008). High-Level Conference on World Food Security: The Challenges of Climate Change and Bioenergy.

[10] FAO (2007). The State of the World’s Animal Genetic Resources for Food and Agriculture.

[11] Cortellari M., Bionda A., Negro A., Frattini S., Mastrangelo S., Somenzi E., Lasagna E., Sarti F.M., Ciani E., Ciampolini R. (2021). Runs of homozygosity in the Italian goat breeds: Impact of management practices in low-input systems. Genet. Sel. Evol..

[12] Bowles D. (2015). Recent advances in understanding the genetic resources of sheep breeds locally-adapted to the UK uplands: Opportunities they offer for sustainable productivity. Front. Genet..

[13] European Commission (2020). Communication from the Commission to the European Parliament, the Council, the European Economic and Social Committee and the Committee of the Regions—EU Biodiversity Strategy for 2030—Bringing Nature Back into Our Lives (COM (2020) 380 final, 20.5.2020). https://www.europarl.europa.eu/doceo/document/TA-9-2021-0277_EN.html.

[14] Kleijn D., Rundlöf M., Scheper J., Smith H.G., Tscharntke T. (2011). Does conservation on farmland contribute to halting the biodiversity decline?. Trends Ecol. Evol..

[15] Cao X., Sun B., Chen H., Zhou J., Song X., Liu X., Deng X., Li X., Zhao Y., Zhang J. (2021). Approaches and research progresses of marginal land productivity expansion and ecological benefit improvement in China. Bull. Chin. Acad. Sci..

[16] Zeder M.A. (2008). Domestication and early agriculture in the Mediterranean Basin: Origins, diffusion, and impact. Proc. Natl. Acad. Sci. USA.

[17] Conversa G., Lazzizera C., Bonasia A., Cifarelli S., Losavio F., Sonnante G., Elia A. (2020). Exploring on-farm agro-biodiversity: A study case of vegetable landraces from Apulia region (Italy). Biodivers. Conserv..

[18] Martinello P., Berardo N. (2004). Qualitative and quantitative pasturelands coenoses in environments with Mediterranean climate of the Apulia region of Italy. Cah. Options Méditerranéennes.

[19] Pulina G., Milán M.J., Lavín M.P., Theodoridis A., Morin E., Capote J., Thomas D.L., Francesconi A.H.D., Caja G. (2018). Invited review: Current production trends, farm structures, and economics of the dairy sheep and goat sectors. J. Dairy sci..

[20] Leroy G., Boettcher P., Joly F., Looft C., Baumung R. (2024). Multifunctionality and provision of ecosystem services by livestock species and breeds at global level. Animal.

[21] Mathew E., Mathew L. (2023). Conservation of landraces and indigenous breeds: An investment for the future. Conservation and Sustainable Utilization of Bioresources.

[22] Pieragostini E., Rubino G., Bramante G., Rullo R., Petazzi F., Caroli A. (2006). Functional effect of haemoglobin polymorphism on the haematological pattern of Gentile di Puglia sheep. J. Anim. Breed. Genet..

[23] Oikonomou D., Vrahnakis M., Yiakoulaki M., Xanthopoulos G., Kazoglou Y. (2023). Grazing as a management tool in Mediterranean Pastures: A meta-analysis based on a literature review. Land.

[24] Petito M., Cantalamessa S., Pagnani G., Pisante M. (2024). Modelling and mapping Soil Organic Carbon in annual cropland under different farm management systems in the Apulia region of Southern Italy. Soil Tillage Res..

[25] Albenzio M., Corbo M.R., Rehman S.U., Fox P.F., De Angelis M., Corsetti A., Sevi A., Gobbetti M. (2001). Microbiological and biochemical characteristics of Canestrato Pugliese cheese made from raw milk, pasteurized milk or by heating the curd in hot whey. Int. J. Food Microbiol..

[26] Farinella D., Nori M., Ragkos A. (2017). Change in Euro-Mediterranean pastoralism: Which opportunities for rural development and generational renewal?. Grassl. Sci. Eur..

[27] Cammerino A.R.B., Biscotti S., De Iulio R., Monteleone M. (2018). The sheep tracks of transhumance in the Apulia region (south Italy): Steps to a strategy of agricultural landscape conservation. Appl. Ecol. Environ. Res..

[28] Associazione Nazionale della Pastorizia (1972). Razze: Consistenza e Distribuzione.

[29] Liberale R. (2000). Pastorizia e Tratturi in Abruzzo. Breve Compendio Storico.

[30] Temerario L., Monaco D., Mastrorocco A., Martino N.A., Cseh S., Lacalandra G.M., Ciani E., Dell’aquila M.E. (2023). New strategies for conservation of Gentile di Puglia sheep breed, an autochthonous capital of millennial tradition in Southern Italy. Animals.

[31] Ceccobelli S., Landi V., Senczuk G., Mastrangelo S., Sardina M.T., Ben-Jemaa S., Persichilli C., Karsli T., Bâlteanu V.-A., Raschia M.A. (2023). A comprehensive analysis of the genetic diversity and environmental adaptability in worldwide Merino and Merino-derived sheep breeds. Genet. Sel. Evol..

[32] Associazione Nazionale della Pastorizia (2023). Internal Statistics.

[33] Muscio A., Albenzio M., Sevi A. (2008). La storia dell’allevamento ovino nel Mezzogiorno. La Valorizzazione delle Razze Ovine Autoctone dell’Italia Meridionale.

[34] Santillo A., Albenzio M. (2008). Influence of lamb rennet paste containing probiotic on proteolysis and rheological properties of Pecorino cheese. J. Dairy Sci..

[35] Albenzio M., Santillo A., Caroprese M., Ruggieri D., Napolitano F., Sevi A. (2013). Physicochemical properties of Scamorza ewe milk cheese manufactured with different probiotic cultures. J. Dairy Sci..

[36] Ciliberti M.G., Santillo A., Marino R., Ciani E., Caroprese M., Rillo L., Matassino D., Sevi A., Albenzio M. (2021). Lamb meat quality and carcass evaluation of five autochthonous sheep breeds: Towards biodiversity protection. Animals.

[37] Maria Sarti F., Lasagna E., Panella F., Lebboroni G., Renieri C. (2006). Wool quality in Gentile di Puglia sheep breed as measure of genetic integrity. Ital. J. Anim. Sci..

[38] Castellana E., Ciani E., Cianci D. (2008). La Razza Altamurana. La Valorizzazione delle Razze Ovine Autoctone dell’Italia Meridionale Continentale.

[39] Claps S., Annicchiarico G., Di Napoli M.A., Paladino F., Giorgio D., Sepe L., Rossi R., Di Trana A. (2016). Native and Non-Native Sheep Breed Differences in Canestrato Pugliese Cheese Quality: A Resource for a Sustainable Pastoral System. Czech J. Food Sci..

[40] della Malva A., Albenzio M., Annicchiarico G., Caroprese M., Muscio A., Santillo A., Marino R. (2016). Relationship between slaughtering age, nutritional and organoleptic properties of Altamurana lamb meat. Small Rumin. Res..

[41] Selvaggi M., Cataldo D. (2015). Genetic analysis of milk production traits in Jonica goats. Small Rumin. Res..

[42] Pieragostini E., Dario C., Bufano G. (1994). Hemoglobin phenotypes and hematological factors in Leccese sheep. Small Rumin. Res..

[43] Dario C., Carnicella D., Dario M., Bufano G. (2008). Genetic polymorphism of β-lactoglobulin gene and effect on milk composition in Leccese sheep. Small Rumin Res..

[44] D’Alessandro A.G., Selvaggi M., Martemucci G. (2016). Fatty acid composition and hedonic ratings of meat from light lambs of leccese breed in relation to slaughter age. Int. J. Adv. Sci. Eng Inform. Techn..

[45] Maiorano G., Kowaliszyn B., D’Alessandro A.G., Martemucci G. (2010). The effect of production system information on consumer expectation and acceptability of Leccese lamb meat. Ann. Food Sci. Technol..

[46] Albenzio M., Caroprese M., Marino R., Muscio A., Santillo A., Sevi A. (2006). Characteristics of Garganica goat milk and Cacioricotta cheese. Small Rumin. Res..

[47] Albenzio M., Santillo A., d’Angelo F., Sevi A. (2009). Focusing on casein gene cluster and protein profile in Garganica goat milk. J. Dairy Res..

[48] Rotondi P., Colonna M.A., Marsico G., Giannico F., Ragni M., Facciolongo A.M. (2018). Dietary supplementation with oregano and linseed in Garganica suckling kids: Effects on growth performances and meat quality. Pak. J. Zool..

[49] Didonna A., Colonna M.A., Renna M., Signore A., Santamaria P. (2022). Atlante Dei Prodotti Agroalimentari Tradizionali di Puglia.

[50] EURISPES (2017). 29° Rapporto Italia. Percorsi di Ricerca Nella Società Italiana.

[51] Guerrero L., Guàrdia M.D., Xicola J., Verbeke W., Vanhonacker F., Zakowska-Biemans S., Sajdakowska M., Sulmont-Rossé C., Issanchou S., Contel M. (2009). Consumer-driven definition of traditional food products and innovation in traditional foods. A qualitative cross-cultural study. Appetite.

[52] European Union (EU) (1996). Commission Regulation (EC) No 1107/96 of 12 June 1996 on the registration of geographical indications and designations of origin under the procedure laid down in Article 17 of Council Regulation (EEC) No 2081/92. Off. J. Eur. Union L.

[53] Santillo A., Albenzio M. (2023). Sensory Profiles of Very Hard Italian Cheeses and Related Varieties. Sensory Profiling of Dairy Products.

[54] Di Cagno R., Upadhyay V.K., McSweeney P.L.H., Corbo M.R., Faccia M., Gobbetti M. (2004). Microbiological, compositional and biochemical characterisation of PDO Canestrato Pugliese cheese. It. J. Food Sci..

[55] Pirisi A., Comunian R., Urgeghe P.P., Scintu M.F. (2011). Sheep’s and goat’s dairy products in Italy: Technological, chemical, microbiological, and sensory aspects. Small Rumin. Res..

[56] Caponio F., Pasqualone A., Gomes T. (2001). Apulian Cacioricotta goat’s cheese: Technical interventions for improving yield and organoleptic characteristics. Europ. Food Res. Techn..

[57] Celano G., Costantino G., Calasso M., Randazzo C., Minervini F. (2022). Distinctive Traits of Four Apulian Traditional Agri-Food Product (TAP) Cheeses Manufactured at the Same Dairy Plant. Foods.

[58] De Pasquale I., Calasso M., Mancini L., Ercolini D., La Storia A., De Angelis M., Di Cagno R., Gobbetti M. (2014). Causal relationship between microbial ecology dynamics and proteolysis during manufacture and ripening of protected designation of origin (PDO) cheese Canestrato Pugliese. Appl. Environ. Microbiol..

[59] Piombino P., Pessina R., Genovese A., Lisanti M.T., Moio L. (2008). Sensory profiling, volatiles and odor-active compounds of Canestrato pugliese PDO cheese made from raw and pasteurized ewes’ milk. Ital. J. Food Sci.

[60] G.U.R.I. (Gazzetta Ufficiale della Repubblica Italiana) Ministero delle Politiche Agricole Alimentari e Forestali Decreto del 23 Maggio 2016. Sedicesima Revisione Dell’elenco Nazionale dei Prodotti Agroalimentari Tradizionali (No. 143 del 21/06/2016). https://www.gazzettaufficiale.it/eli/id/2016/06/21/16A04569/sg.

[61] Sacco A., Sacco D., Casiello G., Ventrella A., Longobardi F. (2011). Investigation on a typical dairy product of the Apulia Region (Italy): The garganico cacioricotta cheese, by means of traditional and innovative physico-chemical analyses. Cheese: Types, Nutrition and Consumption.

[62] Pasqualone A., Caponio F., Alloggio V., Gomes T. (2000). Content of taurine in Apulian Cacioricotta goat’s cheese. Europ. Food Res. Techn..

[63] Pasqualone A., Caponio F., Bilancia M.T. (2003). Effect of milk heating on taurine content and compositional characteristics of Apulian Cacioricotta goat’s cheese. Milchwissenschaft.

[64] Santillo A., Caroprese M., Marino R., Muscio A., Sevi A., Albenzio M. (2007). Influence of lamb rennet paste on chemical and enzymatic characteristics of Pecorino Foggiano cheese. Int. Dairy J..

[65] Santillo A., Albenzio M., Bevilacqua A., Corbo M.R., Sevi A. (2012). Encapsulation of probiotic bacteria in lamb rennet paste: Effects on the quality of Pecorino cheese. J. Dairy Sci..

[66] de Groot R.S., Alkemade R., Braat L., Hein L., Willemen L. (2010). Challenges in integrating the concept of ecosystem services and values in landscape planning, man-agement and decision making. Ecol. Complex..

[67] Chan K.M.A., Guerry A.D., Balvanera P., Klain S., Satterfield T., Basurto X., Bostrom A., Chuenpagdee R., Gould R., Halpern B.S. (2012). Where are cultural and social in ecosystem services? A framework for constructive engagement. BioScience.

[68] Dumont B., Ryschawy J., Duru M., Benoit M., Chatellier V., Delaby L., Donnars C., Dupraz P., Lemauviel-Lavenant S., Méda B. (2019). Associations among goods, impacts and ecosystem services provided by livestock farming. Animal.

[69] MEA (2005). Ecosystems and Human Well-Being.

[70] Merida V.E., Cook D., Ögmundarson Ó., Davíðsdóttir B. (2022). Ecosystem Services and Disservices of Meat and Dairy Production: A Systematic Literature Review. Ecosyst. Serv..

[71] Sardaro R., La Sala P. (2021). New value to wool: Innovative garments for preservation of sheep landraces in Italy. Animals.

